# The Junctional Proteins Cingulin and Paracingulin Modulate the Expression of Tight Junction Protein Genes through GATA-4

**DOI:** 10.1371/journal.pone.0055873

**Published:** 2013-02-07

**Authors:** Laurent Guillemot, Domenica Spadaro, Sandra Citi

**Affiliations:** 1 Department of Molecular Biology, University of Geneva, Geneva, Switzerland; 2 Department of Cell Biology, University of Geneva, Geneva, Switzerland; 3 Institute of Genetics and Genomics in Geneva, University of Geneva, Geneva, Switzerland; Emory University School of Medicine, United States of America

## Abstract

The cytoplamic junctional proteins cingulin and paracingulin have been implicated in the regulation of gene expression in different cultured cell models. In renal epithelial MDCK cells, depletion of either protein results in a Rho-dependent increase in the expression of claudin-2. Here we examined MDCK cell clones depleted of both cingulin and paracingulin (double-KD cells), and we found that unexpectedly the expression of claudin-2, and also the expression of ZO-3 and claudin-3, were decreased, while RhoA activity was still higher than in control cells. The decreased expression of claudin-2 and other TJ proteins in double–KD cells correlated with reduced levels of the transcription factor GATA-4, and was rescued by overexpression of GATA-4, but not by inhibiting RhoA activity. These results indicate that in MDCK cells GATA-4 is required for the expression of claudin-2 and other TJ proteins, and that maintenance of GATA-4 expression requires either cingulin or paracingulin. These results and previous studies suggest a model whereby cingulin and paracingulin redundantly control the expression of specific TJ proteins through distinct GATA-4- and RhoA-dependent mechanisms, and that in the absence of sufficient levels of GATA-4 the RhoA-mediated upregulation of claudin-2 is inhibited.

## Introduction

The apical junctional complex (AJC) of vertebrate epithelial cells comprises tight junctions (TJ) and adherens junctions (AJ), which are critical for tissue barrier functions, cell-cell adhesion and morphogenesis. TJ and AJ contain complexes of transmembrane and cytoplasmic proteins, that are linked to the cytoskeleton, and provide the structural basis for the control of paracellular permeability, adhesion, and scaffolding of membrane proteins [1], [2], [3], [4], [5]. In addition, several TJ and AJ proteins are implicated in the control of gene expression, through different signalling pathways [6], [7], [8].

Claudin-2, a member of the claudin family of transmembrane TJ proteins, is expressed in leaky epithelia and proliferating tissues [9], [10], [11], [12], and its increased expression has been correlated to inflammatory intestinal disease and tumorigenesis [13], [14], [15], [16]. Therefore, investigating the mechanisms that regulate claudin-2 expression may provide essential information about epithelial tissue physiology and pathology. Previously, we showed that depletion of the cytoplasmic AJC proteins cingulin and paracingulin (also known as CGNL1, or JACOP [17], [18]) results in increases in the expression of claudin-2, and in increased RhoA activity in confluent monolayers [19], [20]. Furthermore, cingulin knockout embryoid bodies and epithelial tissues from cingulin knockout mice display increased claudin-2 expression [21], [22]. We found that the increased expression of claudin-2 in cingulin-depleted cells could be reversed by inhibiting RhoA activity, indicating that claudin-2 gene expression is regulated by RhoA [19].

Here, to explore in further detail the redundant functions of cingulin and paracingulin, and their role in controlling the expression of claudin-2 and other TJ protein genes, we generated clonal MDCK cell lines that can be reversibly depleted of both proteins. Surprisingly, we find that in double-KD cells claudin-2 and other TJ proteins show decreased, rather than increased expression, and we identify GATA-4 as the transcription factor that is mechanistically involved in this phenotype, independently of RhoA.

## Results

### Combined depletion of cingulin and paracingulin in MDCK cells results in a decreased expression of claudin-2, ZO-3, and claudin-3

**Figure 1:**
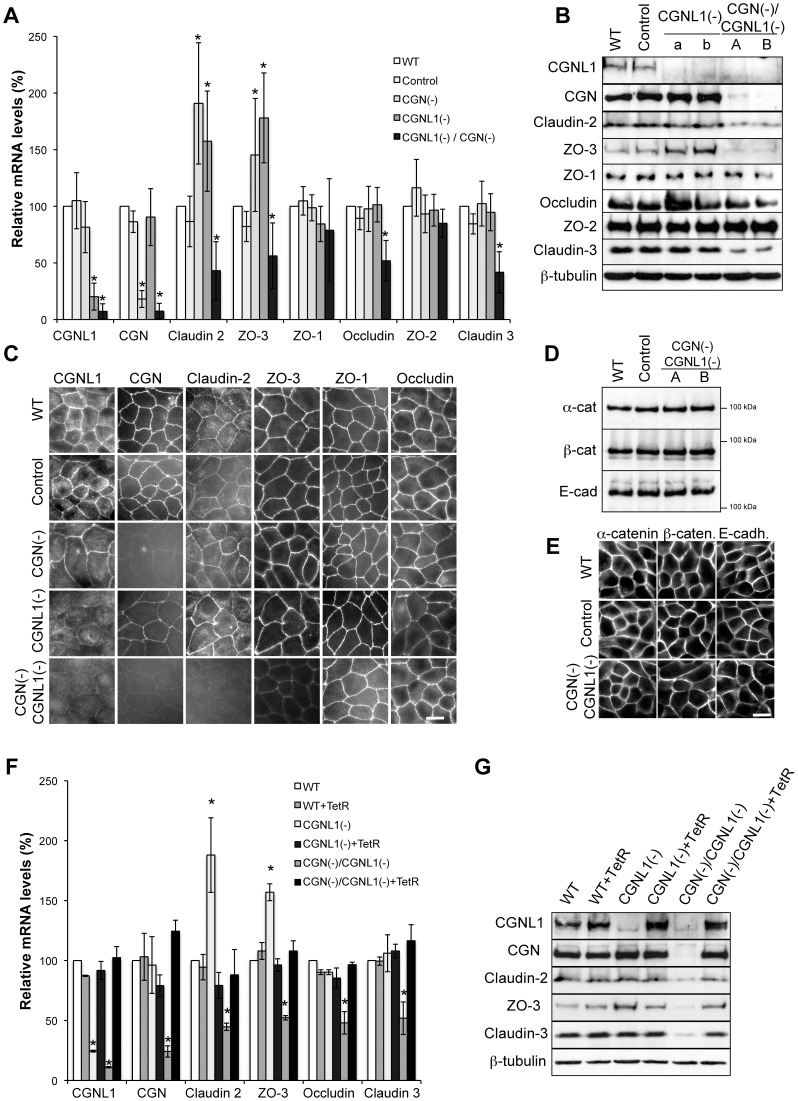
Down-regulation of claudin-2, ZO-3, and claudin-3 in cingulin/paracingulin double knockdown MDCK cells. (**A**) Histogram showing relative mRNA levels, determined by qRT-PCR, for the indicated transcripts, in wild-type MDCK cells (WT), in a MDCK cell clone expressing a control shRNA (Control), in a cingulin single-KD cell clone (CGN(-)), in a paracingulin single-KD cell clone (CGNL1(-)), and in a double-KD cell clone (CGN(-)/CGNL1(-)). The relative mRNA levels were calculated as the ratio of the mRNA in experimental samples versus WT cells (taken as 100%). *p<0.05, compared with mRNA of WT cells. (**B**) Immunoblotting analysis of TJ proteins in WT cells, in a clone expressing a control shRNA (Control), in two independent (a and b) paracingulin single-KD cell clones (CGNL1(-)), and in two independent (A and B) double-KD cell clones (CGN(-)/CGNL1(-)). (**C**) Immunofluorescence localization of CGNL1, CGN, claudin-2, ZO-3, ZO-1 and occludin in WT, control, CGN(-), CGNL1(-) and CGN(-)/CGNL1(-) cells. (**D**) Immunoblotting analysis and (**E**) immunofluorescence localization of α-catenin (α-cat), β-catenin (β-cat), and E-cadherin (E-cad) in wild-type (WT) MDCK cells, in a MDCK cell clone expressing a control shRNA (Control), and in a double-KD (CGN(-)/CGNL1(-)) cell clone. Bar (C) and (E) = 10 µm. Protein loadings in (B) and (D) were normalized by immunoblotting samples with anti-β-tubulin antibodies (shown in (B) only). The behaviour of additional double-KD clonal lines was very similar to the representative one shown here. (**F**) qRT-PCR and (**G**) immunoblotting analysis of WT, paracingulin single-KD (CGNL1(-)) and cingulin/paracingulin double-KD (CGN(-)/CGNL1(-)) cells with or without additional expression of the tetracycline repressor (TetR), which blocks the expression of shRNA. The relative mRNA levels were calculated as the ratio of the mRNA in experimental samples to that in WT cells (taken as 100%). *p<0.05, compared with WT cells. Protein loadings in (G) were normalized by immunoblotting with anti-β-tubulin antibodies.

In cells depleted of either CGN or CGNL1 alone, the levels of claudin-2 mRNA are increased by approximately 2- to 3-fold, when compared to wild-type (WT) cells [19], [20]. This correlates with up-regulated claudin-2 protein expression in CGN-KD, but not CGNL1-KD cells [19], [20]. In CGN-knockout embryoid bodies, claudin-2 mRNA expression is increased 19-fold, with respect to wild-type [21]. Here, to examine in further detail the role of CGN and CGNL1 in the control of claudin-2 expression, we isolated stable MDCK clonal lines that were depleted of both proteins (double-KD cells, CGN(-)/CGNL1(-)). We then used quantitative real-time PCR (qRT-PCR) (Fig. 1A), immunoblot (Fig. 1B), and immunofluorescence (Fig. 1C) analyses to examine the expression and localization of claudin-2 and other TJ proteins. In the double-KD clonal lines the expression levels of CGN and CGNL1 were significantly decreased when compared to wild-type (Fig. 1A–B), and both proteins showed very low or undetectable signal at cell-cell junctions by immunofluorescence (Fig. 1C). Surprisingly, in double-KD cells the expression of claudin-2 was not increased, but instead it was decreased by about 2-fold with respect to wild-type, both at the mRNA (Fig. 1A) and protein (Fig. 1B) levels. The expression of additional TJ proteins was decreased in double-KD cells (Fig. 1A–C). For example, ZO-3 and claudin-3 expression were decreased both at the mRNA and protein level (Fig. 1A–B). ZO-3 and claudin-2 immunofluorescent signals were also notably decreased (Fig. 1C). Occludin expression was reduced significantly at the mRNA level, but not by immunoblot and immunofluorescence (Fig. 1A–C) and therefore its analysis was omitted for subsequent experiments. In summary, CGN and CGNL1 act redundantly to maintain a normal level of expression of claudin-2, claudin-3 and ZO-3, and the depletion of both results in down-regulation of claudin-2, claudin-3 and ZO-3.

Next, we examined the expression and junctional localization of ZO-1 (Fig. 1A–C), and the AJ proteins α-catenin, β-catenin and E-cadherin (Fig. 1D–E). As previously observed in single-KD cells, these proteins were not altered in their expression and localization, as determined by immunoblotting (Fig. 1B, 1D), immunofluorescence (Fig. 1C, 1E) and qRT-PCR (data not shown). The expression of additional TJ and signaling proteins, including DbpA/ZONAB, Par-3 and ZO-2 was also examined, but was not significantly changed in double-KD cell clones (data not shown). This indicated that the combined depletion of CGN and CGNL1 does not affect the overall molecular structure and organization of the AJC, but only selectively influences the expression of specific proteins.

Finally, to exclude the possibility that the phenotype was due to clone-dependent variations, we rescued the expression of CGN and CGNL1 in double-KD cells by exogenously expressing the Tetracycline repressor (TetR). Following re-expression of CGN and CGNL1, the levels of expression of claudin-2, ZO-3, and claudin-3 returned to normal, both at the mRNA (Fig. 1F) and protein (Fig. 1G) levels.

### The transcription factor GATA-4 is mechanistically involved in the regulation of the expression of claudin-2 and other TJ proteins by cingulin and paracingulin

**Figure 2:**
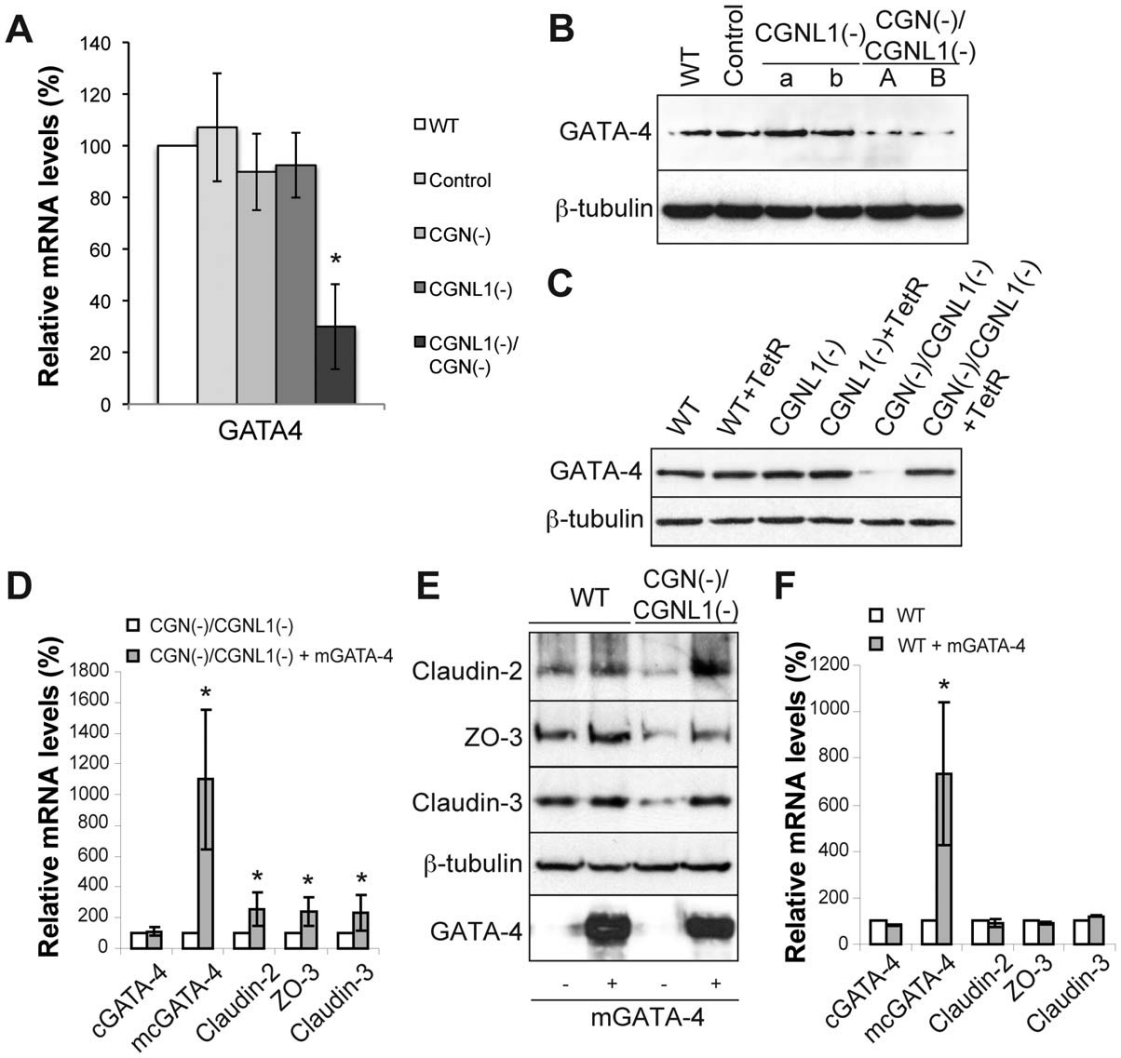
Decreased GATA-4 levels cause reduced expression of claudin-2, ZO-3 and claudin-3 in double-KD cells. (**A**) qRT-PCR analysis of the mRNA levels of GATA-4 in the different MDCK cell clones. *p<0.05, compared with WT cells (taken as 100%). (**B**) Immunoblot analysis of GATA-4 protein levels in wild-type MDCK cells (WT), in a MDCK cell clone expressing a control shRNA (Control), in two independent (a and b) paracingulin single-KD cell clones (CGNL1-), and in two independent (A and B) double-KD cell clones (CGN(-)/CGNL1(-)). Protein loadings were normalized by immunoblotting with anti-β-tubulin antibodies. (**C**) Immunoblot analysis of protein levels of GATA-4 in WT cells, paracingulin single-KD cells, and cingulin/paracingulin double-KD MDCK cell clones, either in the absence or in the presence (+TetR) of exogenously expressed tetracycline repressor. The decreased expression of GATA-4 is rescued by expression of TetR. (**D**) and (**F**) Histograms showing mRNA levels (determined by qRT-PCR) of canine GATA-4 (cGATA-4), mouse+canine GATA-4 (mcGATA-4), claudin-2, ZO-3 and claudin-3 either in double-KD MDCK cells (E) or in WT cells (G), without (white columns) or with (grey columns) the exogenous expression of mouse GATA-4 (m-GATA-4). Mouse GATA-4 was used for the rescue because a canine GATA-4 cDNA was not available, and oligos used for GATA-4 RT-PCR were either canine-, or mouse+canine-specific. (**E**) Immunoblotting analysis of lysates of the same cell lines shown in (D) and (F), using antibodies against claudin-2, ZO-3, claudin-3, and GATA-4 (mouse-specific antibodies, which do not cross-react with canine GATA-4). Expression of exogenous GATA-4 in double-KD cells rescues mRNA and protein levels of claudin-2, ZO-3, claudin-3, while exogenous expression of mouse GATA-4 in WT cells has little or no effect on the levels of endogenous mRNA and protein.

To dissect the molecular mechanism through which CGN and CGNL1 redundantly control claudin-2 and ZO-3 expression, we focused on the transcription factor GATA-4, based on the premise that GATA-4 was previously shown to promote claudin-2 expression in intestinal epithelia [23]. qRT-PCR and immunoblotting showed that there were significant decreases in the mRNA (Fig. 2A) and protein (Fig. 2B) levels of GATA-4 in double-KD cells, when compared to either CGN or CGNL1 single-KD cells, or to wild-type or control cells. In contrast, single depletion of either CGN or CGNL1 alone did not significantly affect the expression of GATA-4 (Fig. 2A–B). This phenotype was not due to clone-dependent variations, since the expression of GATA-4 was rescued by exogenous expression of the Tetracycline repressor (TetR) both at the protein and mRNA levels (Fig. 2C, and data not shown).

To test the hypothesis that decreased levels of GATA-4 are mechanistically implicated in the decreased expression of claudin-2, ZO-3 and claudin-3 in double-KD cells, we asked whether we could rescue the phenotype by overexpression of exogenous GATA-4. Strikingly, exogenous expression of mouse GATA-4 resulted in more than doubled expression levels of mRNA (Fig. 2D) and protein (Fig. 2E) for claudin-2, ZO-3, and claudin-3, when compared to untreated double-KD cells. On the other hand, overexpression of mouse GATA-4 in wild-type cells did not significantly increase either mRNA or protein levels for either TJ proteins, or endogeneous GATA-4 (Fig. 2E–F). Also, although the exogenous expression of GATA-4 resulted in much higher total levels of GATA-4 with respect to control cells (Fig. 2D–F), the expression of claudin-2, ZO-3 and claudin-3 did not increase beyond wild-type levels. These results indicate that in kidney epithelial cells a) GATA-4 is mechanistically involved in the regulation of transcription of claudin-2, claudin-3 and ZO-3, b) the decreased expression levels of these proteins in double-KD cells can be accounted for by the decreased GATA-4 levels, and c) in wild-type cells GATA-4-dependent control of claudin-2, ZO-3 and claudin-3 expression is maximal at physiological concentrations of GATA-4, and cannot be further up-regulated by increasing GATA-4 expression.

### In double-KD CGN(-)/CGNL1(-) cells there is increased RhoA activity, and GATA-4 regulates the expression of claudin-2, ZO-3 and claudin-3 independently of RhoA

In single CGN and CGNL1 KD cells RhoA activity is increased at confluence, and the increased RhoA activation is involved in the up-regulation of claudin-2 expression, since inhibition of RhoA activity rescues this phenotype [19], [20]. Therefore, we tested whether in cingulin/paracingulin double-KD cells there could be a decreased, rather than an increased RhoA activity at confluence, which might explain the decreased expression of claudin-2.

**Figure 3:**
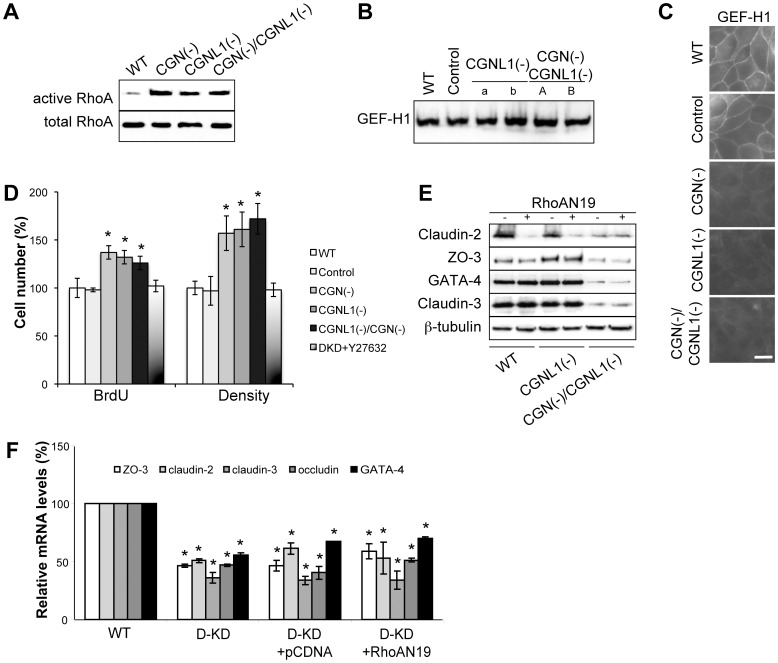
Increased RhoA activity does not account for the phenotype of double-KD cells. (**A**) RhoA activation assay (GST pulldown assay using the rhotekin-binding-domain, see [19]), showing that RhoA activity is increased at confluence in double-KD cells, similarly to both CGN and CGNL1 single-KD cells. (**B**) Immunoblot and (**C**) immunofluorescence analysis of the expression and localization of GEF-H1 in the indicated clonal cell lines. Protein levels of GEF-H1 are not altered in double-KD cells, but the junctional localization of GEF-H1 in confluent cells is decreased in double-KD cells, similar to single-KD cells. Bar, 10 µ m. (**D**) Histogram displaying cell proliferation (BrdU-incorporating cells) and cell density (cell numbers after 7 days growth at confluence) in wild-type, control, CGN and paracingulin single-KD cell clones, and cingulin/paracingulin double-KD cell clones, these latter either in the absence or in the presence of the ROCK kinase inhibitor Y27632 (50 µM). *p<0.05, compared with WT cells (taken as 100%). (**E**) Immunoblotting and (**F**) qRT-PCR analysis of the mRNA and protein levels of claudin-2, ZO-3, GATA-4 and claudin-3 in WT, paracingulin single-KD, and cingulin/paracingulin double-KD cells, without or with the exogenous expression of dominant-negative RhoA (RhoAN19). In (F) data are also shown for double-KD clones expressing only the vector (pcDNA). *p<0.05, compared with WT cells (taken as 100%).

Confluent cells were lysed, and RhoA activity was measured by a GST pulldown assay [19]. The RhoA activity in double-KD cells was increased, similarly to single-KD cells (Fig. 3A). The levels of expression of the RhoA activator GEF-H1, when assessed by immunoblotting (Fig. 3B) and RT-PCR (not shown), were not altered in double-KD cells. Moreover, increased RhoA activity in double-KD cells correlated with a decreased junctional accumulation of GEF-H1, as previously observed in single-KD cells [20] (Fig. 3C). Increased RhoA activity was correlated with increased cell proliferation and confluent density both in CGNL1 single-KD cells, and in cingulin/paracingulin double-KD cells, as determined by a BrdU incorporation assay, and by counting cells after seven days of confluent growth (Fig. 3D). Furthermore, inhibition of the activity of ROCK kinases, downstream effectors of active RhoA, through the inhibitor Y27632, resulted in a complete rescue of the phenotype in single-KD and double-KD cell lines (Fig. 3D), indicating that the increased cell proliferation and density of double-KD cells is mediated through a RhoA- and ROCK-kinase-dependent pathway.

To check that increased RhoA activity in double-KD cells is not involved in the GATA-4-dependent regulation of gene expression, we expressed a dominant-negative RhoA mutant either in WT, or CGNL1 single-KD cells, or in cingulin/paracingulin double KD cells. Immunoblot analysis confirmed that inhibition of RhoA activity in wild-type and CGNL1 single-KD cells resulted in decreased claudin-2 expression (Fig. 3E) [19], [20]. In contrast, expression of dominant-negative RhoA in double-KD cells neither further decreased nor rescued the decreased expression of ZO-3, claudin-2, claudin-3 and GATA-4, both at the protein (Fig. 3E) and mRNA levels (Fig. 3F). Taken together, these observations indicate that increased RhoA activity is not implicated in the decreased expression of GATA-4, and that both RhoA inhibition and decreased GATA-4 expression can lead to decreased expression of claudin-2, ZO-3, and claudin-3. However, when GATA-4 levels are low, these TJ proteins are expressed at low levels, independently of RhoA activation.

## Discussion

The apical junctional complex (AJC) functions as a platform that integrates signals from the intra- and extra-cellular environments, to modulate gene expression, cytoskeletal organization and epithelial morphogenesis. A few pathways that link junctional proteins to transcription factors and modulation of specific target genes have been described [6], [7]. Previously, we characterized cingulin and paracingulin as AJC proteins implicated in the regulation of the expression of claudin-2 in cultured cells, through RhoA signaling. Specifically, in either cingulin or paracingulin single-KD cells expression of claudin-2 mRNA was increased, and this increase could be reversed by inhibiting RhoA activity [19], 20. The increase of claudin-2 expression in cingulin-KD cells is physiologically relevant, since it has also been observed in epithelia of cingulin knockout mice [22]. Here, based on the unexpected observation that in cells depleted of both cingulin and paracingulin the expression of claudin-2 and other TJ proteins is decreased, independently of RhoA, we identify a new molecular mechanism linking the AJC to control of gene expression, through the transcription factor GATA-4.

Differentiation of epithelial cells is associated with transcriptional modulation of the expression of many junctional proteins, including cingulin and other TJ proteins [24]. However, the role of specific transcription factors in regulating the expression of TJ proteins is not fully understood. Specifically, nothing was known until the present study about transcription factors implicated in the regulation of the claudin-3 and ZO-3 genes, and GATA-4 was implicated in the regulation of claudin-2 expression only in intestinal epithelia [23]. Our work identifies for the first time GATA-4 as a regulator of the expression of not only claudin-2, but also claudin-3 and ZO-3, in kidney epithelial cells. Interestingly, the analysis of claudin-2 and ZO-3 promoter sequences shows the presence of consensus site for GATA-factor binding (A/TGATA(A/G), suggesting that GATA-4 may directly act on these promoters. However, the claudin-3 promoter is missing GATA-factor consensus binding sites, suggesting that its regulation by GATA-4 is indirect. The cingulin gene is also a potential GATA-factor target, since its promoter displays two GATA-binding consensus sites. Cingulin is also a potential target of HNF4-α [25], a transcription factor implicated in the up-regulation of the expression of several TJ proteins [26]. Additional transcription factors that have been implicated in the modulation of TJ protein expression are snail, for occludin and claudins [27], [28], [29], and Tcf and FoxO1 for claudin-5 [30]. The transcriptional regulation of claudin-2 has been investigated by other studies. The claudin-2 promoter is responsive to the TCF-4/β-catenin complex, and to Cdx-1, Cdx-2 and HNF-1 transcription factors [31], [32]. Furthermore, the localization of the transcription factors Cdx-2, HNF-1-α, and GATA-4 in the developing human intestine suggest that all these factors contribute to regulating claudin-2 expression, and that optimal claudin-2 expression in the intestine requires GATA-4 [23]. Our observation that GATA-4 levels are critical to control the expression of claudin-2, and also claudin-3 and ZO-3, in MDCK cells reveals a new role of GATA-4 in kidney epithelial cells. It will be interesting to evaluate the possible role of GATA-4 in the increased expression of claudin-2 in different human epithelial pathologies, including inflammatory bowel disease and cancer.

**Figure 4:**
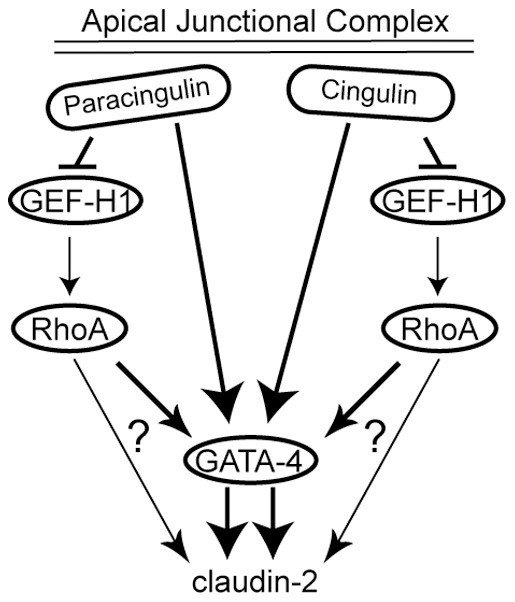
Hypothetical schematic model for the regulation of claudin-2 expression in MDCK cells. GATA-4 is required for optimal transcription of claudin-2 (and other TJ proteins, not shown for simplicity) (double arrow in scheme). Cingulin and paracingulin redundantly control the mRNA and protein expression of GATA-4, and independently contribute to the down-regulation of active RhoA in confluent cells. Active RhoA increases claudin-2 expression, either through GATA-4, and/or other, unknown mechanisms (question marks), but is ineffective when GATA-4 is down-regulated.

GATA-4 is a key transcription factor for the differentiation of primitive endoderm, visceral and parietal endoderm, and endodermal derivatives, such as endocardium, intestine and gonads [33], [34], [35], [36], [37]. However, little is known about the mechanisms regulating GATA-4 expression, especially in epithelial cells. Stat-3 and Ras pathways have been implicated in the control of GATA-4 expression in embryonal carcinoma and ES cells [38], [39], and experiments using GATA-6 knockout and chimeric mice suggest a cross-regulation between GATA-6 and GATA-4 [40]. Interestingly, experiments on cardiac myocytes identified GATA-4 as a nuclear mediator of RhoA signaling [41], [42]. However, GATA-4-dependent transcription was decreased in our CGN(-)/CGNL1(-) double-KD cells, despite the increased RhoA activity. This apparent discrepancy can be explained by the observation that GATA-4 levels were decreased in double-KD cells, and the possibility that Rho signaling becomes ineffective in the absence of physiological levels of GATA-4. So, our results do not exclude the possibility that GATA-4 may function as a mediator of RhoA signaling in epithelial cells, and suggest that in double-KD cells a RhoA-independent pathway, which is regulated redundantly by cingulin and paracingulin, overrides the effects of increased RhoA activation on claudin-2 expression. Taken together, our findings support a model (Fig. 4) where the expression of claudin-2 and other TJ protein genes requires physiological levels of GATA-4, and can be further increased by increasing RhoA activity. Increased RhoA activity might promote claudin-2 expression both in a GATA-independent and –dependent manners (Fig. 4). However, in cingulin/paracingulin double-KD cells, the increased RhoA activity is not sufficient to maintain claudin-2 expression, due to the decreased levels of GATA-4.

In summary, our results show that in renal epithelial cells depletion of both cingulin and paracingulin results in reduced expression of GATA-4, independently of RhoA, leading to a down-regulation of the expression of claudin-2 and other TJ protein genes. This evidence supports the concept that the molecular composition of the AJC plays a critical role in signaling and in modulation of gene expression. Precisely how GATA-4 promotes the transcription of TJ protein genes in epithelial cells, and which signaling pathway(s) mediate the cingulin/paracingulin-dependent down-regulation of GATA-4 are interesting questions to be adressed in future studies.

## Materials and Methods

### Ethics statement. N/A

#### Antibodies and plasmids

Commercial antibodies were obtained against GATA-4 (Santa Cruz), and claudin-3 (Invitrogen). Plasmid encoding mouse GATA-4 (in pcDNA) was a kind gift from T. Evans (Albert Einstein College of Medecine, New York, USA) [35]. Additional constructs (TetR, RhoAN19, GST-rhotekin, etc) and antibodies were described previously [19], [20], [21], [43].

#### Cell culture, transfection and other techniques

shRNA hairpins were cloned in the vector pTER [44](either the original vector, or one where we replaced the zeocin resistance cassette was with a hygromycin resistance cassette, pTER-hygro) to target the following sequences: CGN: 5′-AGAGCATGTTCCAGAAGAA, CGNL1: 5′-CGGAAAGTCAACCTGGTCT-3′; and Control, 5′TCTACGACCCTTCTTCCAT-3′. Transfections were carried out using Lipofectamine2000, and MDCK II cell clones stably expressing shRNA were isolated by cloning rings in antibiotic selection (0.6 mg/ml zeocin (CGN(-)), 0.2 mg/ml hygromycin (CGNL1(-)). To prepare double-KD cell clones, the CGN(-) clone A [19] was transfected with pTER-hygro containing the shRNA that silences CGNL1 expression, and stable clones were selected in a medium containing hygromycin. Cell culture, RhoA activation pulldown assays, cell fractionation, immunoblotting, immunofluorescence, and were as described previously [19], [20], [43], [45]. All experiments were carried out at least in triplicate, and histogram values show means ± standard deviation (S.D.). Values were considered statistically significant (*) when p<0.05 between experiments (Student's *t* tests). For immunoblots and immunofluorescence data, one representative example out of 2–3 independent experiments is shown.

#### Quantitative RT-PCR

mRNA levels were analyzed by SYBR green-based real-time PCR using primers described previously for CGN, CGNL1, claudin-2, ZO-3, ZO-1, occludin, and GEF-H1 [19], [20], [21]. New primers were designed for the following transcripts: claudin-3 forward, 5′-AGT CGT ACA CCT TGC ACT GCA-3′; claudin-3 reverse, 5′-GAG GGC CTG TGG ATG AAC TG-3′; canine (c) GATA-4 forward, 5′-TCCACCCAGTCCTCTCGG-3′; canine (c) GATA-4 reverse, 5′-GGCGACTGGCTGACAGAAG-3′; mouse/canine (mc) GATA-4 forward, 5′-CACTACCTGTGCAATGCCTG-3′; mouse/canine (mc) GATA-4 reverse, 5′-ATGAGGGGCCGGTTGATGC-3′.

#### Promoter sequence analysis

Sequences of the promoter regions of TJ proteins were obtained from genecards.org and switchgeargenomics.com, and analyzed with SerialCloner2–5 software.
